# Sparse Image Registration-Based Marker-Free Hand Acupoint Localization Using TCM Template Queries

**DOI:** 10.3390/s26175552

**Published:** 2026-09-01

**Authors:** Shujian Zhang, Shuyue Zhang, Chi Zhang, Jianqing Peng

**Affiliations:** 1 School of Intelligent Systems Engineering, Shenzhen Campus of Sun Yat-sen University, Shenzhen 518107, China; zhangshj59@mail2.sysu.edu.cn (S.Z.); zhangshy233@mail2.sysu.edu.cn (S.Z.); zhangch287@mail2.sysu.edu.cn (C.Z.); 2 Guangdong Provincial Key Laboratory of Fire Science and Technology, Guangzhou 510006, China

**Keywords:** sensor-based medical imaging, deep learning, hand acupoint localization, marker-free localization, sparse image registration, Transformer, traditional Chinese medicine

## Abstract

Deep learning for sensor-based medical imaging provides a non-contact and data-driven route for anatomical surface analysis and personalized traditional Chinese medicine (TCM) applications. However, accurate hand-acupoint localization from camera-acquired hand images remains challenging because expert-annotated acupoint datasets are limited and inter-subject anatomical variations are significant. This paper proposes a marker-free hand-acupoint localization method based on deep feature correspondence learning and sparse image registration. The task is formulated as template-to-target correspondence estimation, in which expert-annotated acupoints in a TCM template image are used as query points and mapped to a target hand image acquired by an optical imaging sensor. A Transformer-based architecture is employed to correlate multi-scale image features, and an uncertainty-aware matching formulation is used to estimate both acupoint positions and unreliable matches. Unlike conventional keypoint detection networks, the proposed method exploits TCM template priors and reduces the dependence on dense target-image acupoint annotations. The constructed dataset contains 1400 images from 378 participants. Participant-level partitioning was performed before image-pair generation, yielding a held-out test set of 38 participants (140 images). Palm and dorsal-hand views were evaluated separately against keypoint-detection baselines and COTR. On this participant-independent internal test set, the proposed method achieved AAPE values of 18.42 pixels for palm images and 12.60 pixels for dorsal-hand images, while reducing inference time from 11,000 ms for COTR to 600 ms. These results indicate the feasibility of template-guided image-based hand-acupoint localization with reference to expert annotations.

## 1. Introduction

Sensor-based medical imaging increasingly relies on deep-learning algorithms to transform camera-acquired images into quantitative anatomical information. TCM has undergone increasing internationalization in recent years [1]. Compared with contact-based or marker-based measurement, optical hand-image sensing enables non-contact, low-cost, and easily deployable acquisition of anatomical surface features. In image-based traditional Chinese medicine (TCM), hand images captured by optical sensors can provide visual evidence for more consistent image-based acupoint annotation and may reduce operator-dependent variability. Acupoint-related therapies require accurate localization of anatomical acupoints [2,3,4], but conventional manual localization relies heavily on experienced practitioners and extensive clinical training. Therefore, developing a deep-learning-based visual sensing framework for hand-acupoint localization is important for sensor-based medical image analysis and personalized TCM-related applications. The intended use of the present method is image-based localization assistance for research annotation, educational guidance, and potential robotic-assistance workflows.

With the rapid development of deep learning and visual sensing, increasing attention has been paid to vision-based acupoint identification from sensor-acquired images [5,6,7,8,9,10,11]. Current acupoint-perception paradigms can be broadly divided into marker-based and marker-free approaches. Marker-based methods attach fiducial or adhesive markers to the body surface and then localize these markers using feature matching or object detection algorithms. Although these methods can simplify visual perception, the markers may occlude the target skin area, introduce additional preparation steps, and reduce the naturalness of image acquisition. Marker-free localization is therefore more suitable for scalable, non-invasive, and camera-based medical imaging scenarios.

**Figure 1:**
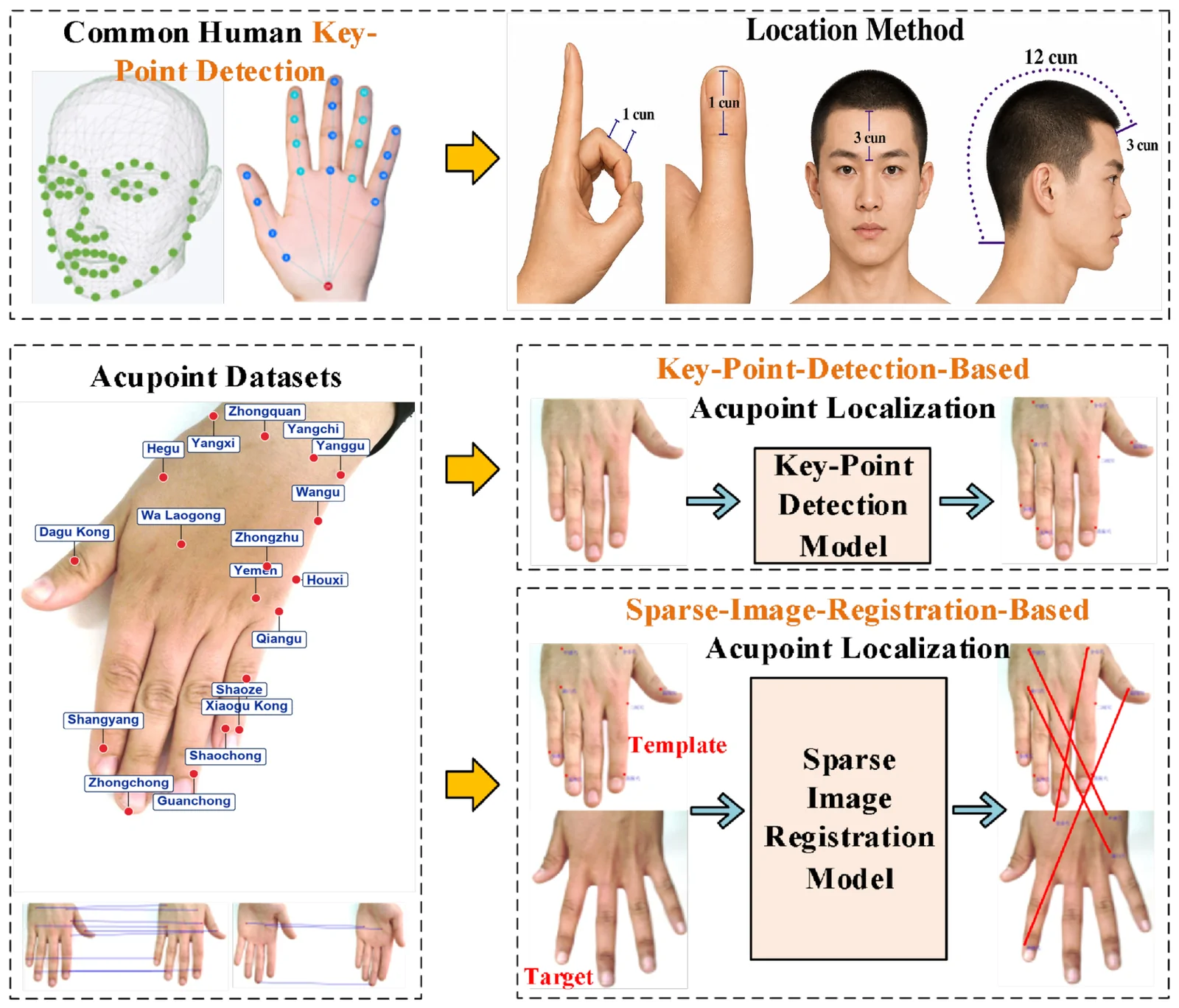
Overview of marker-free acupoint localization methods. Anatomical keypoint detection and TCM proportional measurements support indirect localization; direct keypoint detection predicts acupoints from target hand images; and sparse image registration transfers expert-annotated template acupoints to a target image.

Marker-free acupoint localization is typically formulated as a keypoint detection task. However, acupoint keypoint datasets are scarce and difficult to acquire because annotation requires the expertise of senior acupuncturists. In the absence of sufficient data, training a deep-learning model for direct acupoint localization becomes challenging. To address this limitation, researchers first extract common anatomical key points with off-the-shelf networks and then use TCM prior knowledge to infer acupoint positions indirectly. Traditional acupuncture documents four widely used locating methods: the convenient-point method, the surface-anatomical-landmark method, the finger-cun method, and the bone-proportional-cun method. These methods treat joints, the navel, facial features, and other anatomical landmarks as references and define a proportional anatomical unit called “cun” (Figure 1). In this context, “cun” is subject-specific and should not be regarded as a fixed physical length. Recently, several studies have explored direct acupoint localization using dedicated acupoint datasets. Sun et al. [9] localized hand acupoints using the HRNet model, while Yuan et al. [10] and Malekroodi et al. [11] employed YOLOv8-based or pose-estimation-based methods to identify acupoints on the face and hand. Nevertheless, the limited scale of existing acupoint datasets still constrains model performance. Zhang et al. [5] combined keypoint information and acupoint theory to propose a CNN-based human acupoint detection method. Wang et al. [6] proposed a lightweight acupoint localization method to improve deployment efficiency.

Acupoint localization can also be formulated as a sparse image-registration task whose goal is to establish correspondences between features, such as key points and distinctive regions, in a template image and a target image [12], as illustrated in Figure 1. SuperPoint [13] was the first network to jointly extract key points and descriptors for sparse registration. COTR [14] is a Transformer-based sparse-registration model: it employs a shared feature-extraction backbone to obtain feature maps from two images and then uses self-attention and cross-attention mechanisms to learn feature correspondences. Key points in the template image are treated as queries, and the network predicts their corresponding locations in the target image. To handle scale discrepancies between images, COTR adopts an iterative refinement framework that repeatedly crops local patches and re-registers them, which improves accuracy but incurs redundant computation. To improve efficiency, ECO-TR [15] introduces a multi-scale Transformer registration pipeline that progressively refines matches from coarse to fine levels without repeatedly extracting image features. For hand-acupoint localization, an expert-annotated TCM template can serve as the template image, and target acupoints can then be mapped from the template to a sensor-acquired hand image. Compared with direct keypoint detection, this formulation allows multiple same-view image combinations with shared annotated acupoints to be converted into sparse-registration samples, thereby increasing the effective amount of correspondence supervision available from a limited set of annotated images.

In summary, existing marker-free hand-acupoint localization research has primarily focused on keypoint detection, which can be categorized into indirect and direct schemes. The former relies on traditional acupoint locating rules and is sensitive to inter-subject variations in surface anatomy. The latter is constrained by the scarcity of hand-acupoint datasets; small corpora are insufficient to train models that are both highly accurate and broadly generalizable. To alleviate dependence on large-scale datasets, this paper proposes a deep-learning-based marker-free hand-acupoint localization approach that integrates optical hand-image sensing, TCM prior knowledge, and sparse image registration. The main contributions of this paper are as follows:

1. A deep-learning-based marker-free hand-acupoint localization framework is proposed for sensor-based medical imaging applications. By leveraging a Transformer architecture, the model establishes feature correspondences between a TCM-template image and a camera-acquired target hand image, enabling accurate acupoint querying without attached markers.
2. A TCM-template-query sparse registration strategy is developed to reduce dependence on large-scale acupoint annotations. The expert-annotated template provides prior anatomical knowledge, and the target locations are estimated through template-to-target correspondence prediction.
3. An efficient multi-scale feature-map refinement module is introduced to improve registration accuracy while reducing redundant feature extraction. A physician-annotated hand-acupoint dataset containing palm and dorsal-hand images is constructed for model training and internal evaluation, and the annotated images are further converted into sparse image-registration pairs.

The remainder of this paper is organized as follows. Section 2 introduces the materials and methods, including the sparse-registration framework, the TCM-template query model, and the dataset construction procedure. Section 3 presents the experimental results and comparisons with keypoint detection and sparse-registration baselines. Section 4 discusses the advantages, limitations, and future extensions of the proposed method. Section 5 concludes the paper.

## 2. Materials and Methods

### 2.1. Marker-Free Hand Acupoint Localization Framework Based on Sparse Image Registration

The accuracy of hand acupoint localization based on traditional Chinese medical theory is inherently limited by inter-subject variability in surface anatomy. Moreover, hand-acupoint datasets are scarce and typically small, so keypoint-detection models trained on them suffer from poor stability and limited generalization. To exploit TCM prior knowledge while relaxing the demand for large datasets, this paper proposes a hand acupoint localization framework built upon sparse image registration.

In this study, acupoint localization is cast as a sparse image-registration task. Specifically, the TCM template image is defined as an image whose acupoints have been precisely annotated by senior TCM practitioners; hence, the positions of the target acupoints in this image are treated as prior knowledge. The target hand image is the image acquired by an optical camera. The sparse-registration model actively establishes feature correspondences for hand acupoints between the two images and uses these correspondences to estimate the acupoint locations in the target hand image, thereby boosting localization accuracy.

**Figure 2:**
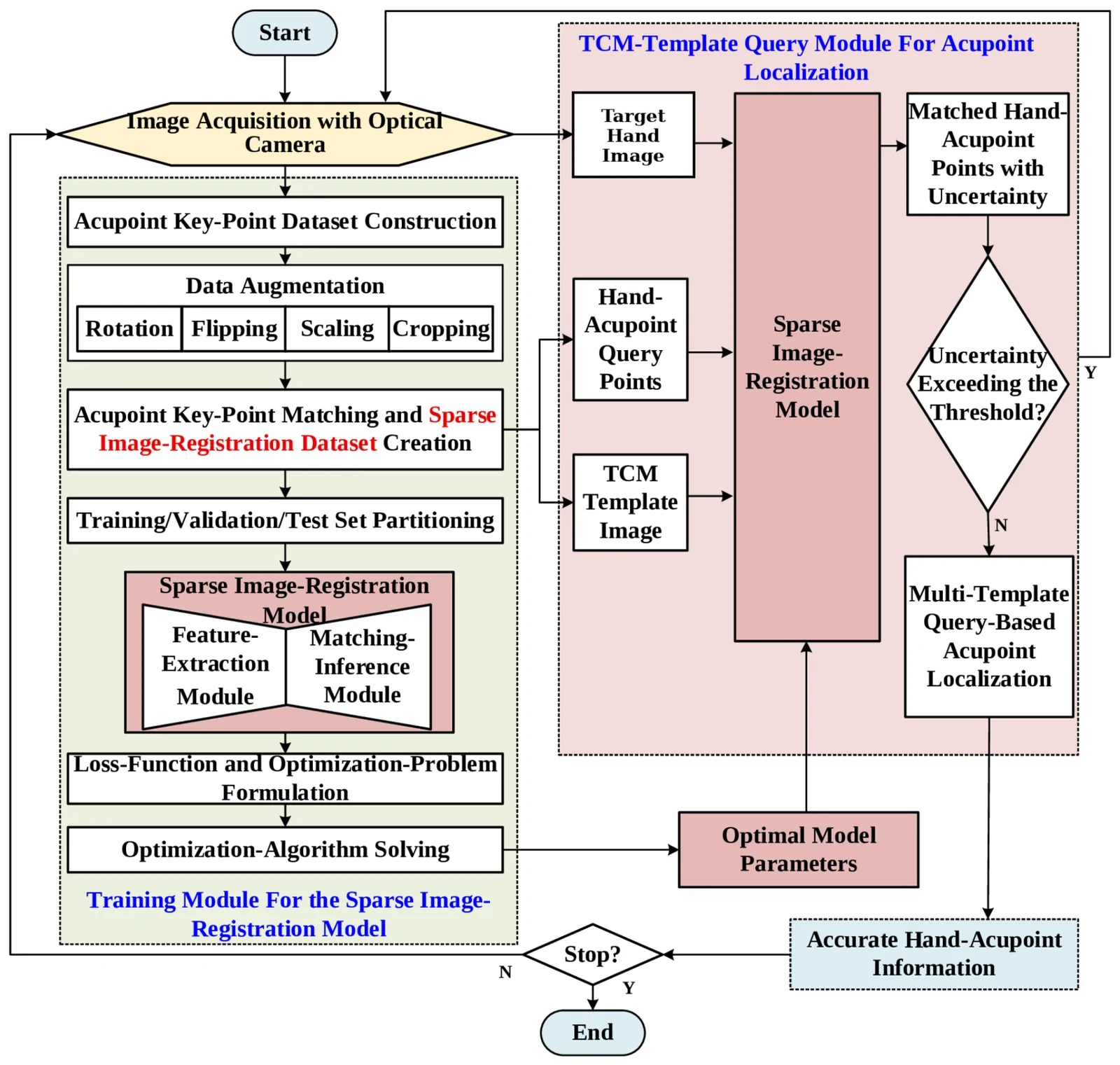
Marker-free hand acupoint localization framework via sparse image registration.

As shown in Figure 2, the proposed marker-free acupoint-perception framework based on sparse image registration contains two main components. First, in the training module for the sparse image-registration model, the model is trained offline before practical deployment. Under the guidance of experienced TCM physicians, hand images are annotated and cleaned to create an acupoint keypoint dataset. Data-augmentation techniques are then applied, and image pairs are formed from the dataset. Acupoints visible in both images are linked to construct a sparse-registration dataset for acupoints, which is used for training and validation. Finally, pretrained weights are loaded, the corresponding loss functions and optimization objectives are defined, and an optimizer is employed to obtain the model parameters.

Second, in the acupoint localization module via TCM-template querying, a camera-acquired target hand image is used as the input. The TCM template image and the reference positions of the target acupoints are loaded from the sparse-registration dataset. The optimal model parameters are then loaded, and both images, together with the query acupoints, are fed into the sparse-registration model to obtain acupoint locations in the target hand image along with their uncertainties.

### 2.2. TCM-Template-Based Hand-Acupoint Localization Model

Let $I_{\mathrm{tmp}}$ and $I_{q}$ denote the TCM template (reference) image and the target hand image, respectively, and let *K* be the number of hand acupoints. The annotated acupoints in the template image are used as query points. For the *k*-th acupoint (k=1,2,…,K), $q_{k}$ denotes the query coordinate in the template image, $p_{k}$ denotes its ground-truth correspondence in the target image, and ${\hat{p}}_{k,i}$ denotes the predicted target coordinate at refinement stage i∈{c,m,f}. The implemented supervised correspondence is therefore a one-way template-to-target matching process.

**Figure 3:**
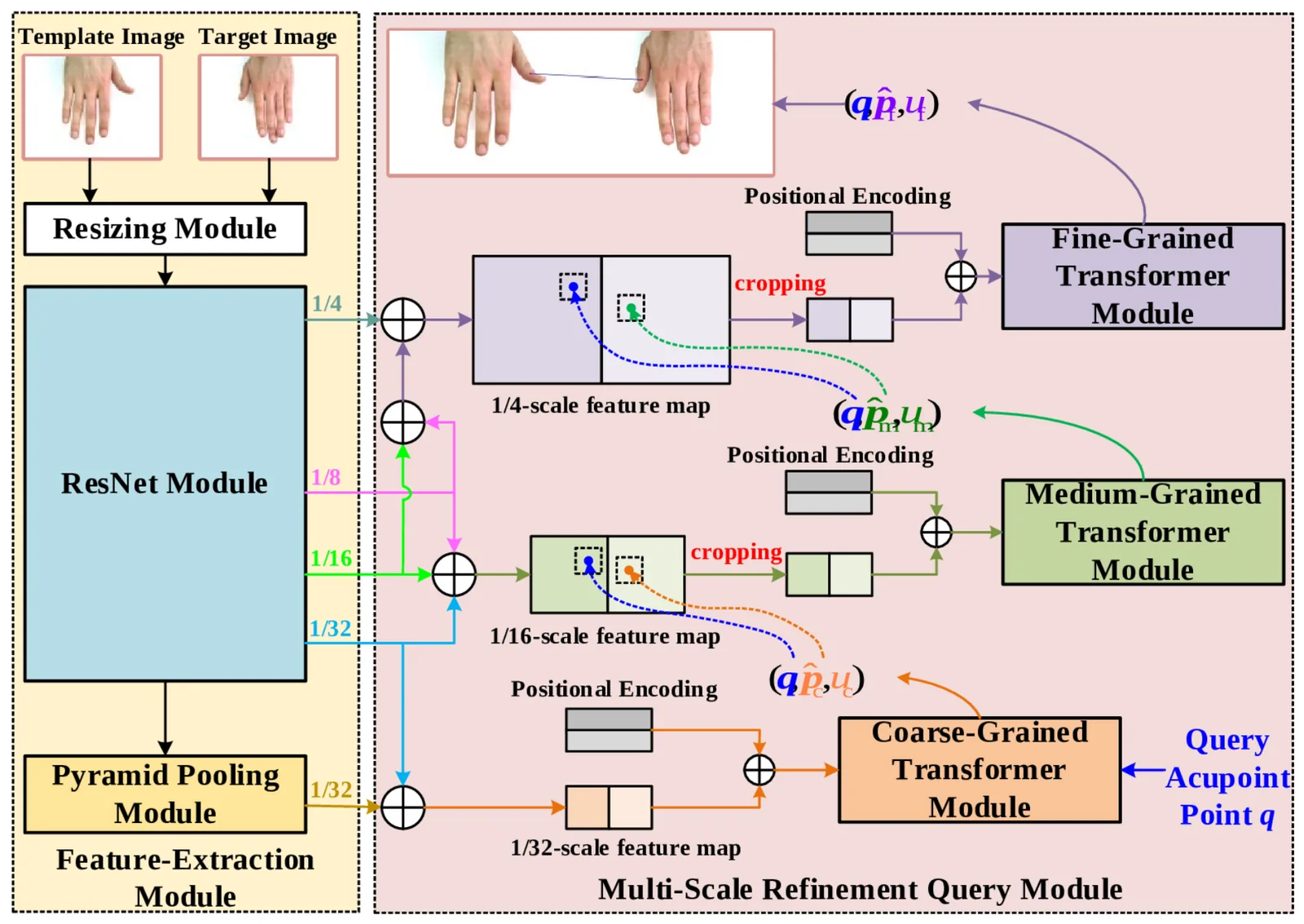
TCM-template-based acupoint localization model.

The TCM-template-based acupoint localization model is illustrated in Figure 3 and consists of a feature-extraction module and a multi-scale refinement query module. The feature-extraction module is responsible for producing feature maps of both the TCM template image and the target hand image. It first resizes $I_{\mathrm{tmp}}$ and $I_{q}$ to the same spatial resolution, then feeds them into a weight-shared feature-extraction network. This network is composed of a ResNet backbone followed by a pyramid pooling module: the ResNet extracts multi-scale feature maps, while the pyramid pooling module performs pooling and aggregation at various scales on the deepest feature maps, thereby enhancing the network’s ability to capture global contextual information. Specifically, we use a custom ResNet50-FPN backbone with Bottleneck blocks [3,4,6,3]. The ResNet stem consists of a 7×7 stride-2 convolution followed by 3×3 stride-2 max pooling, without dilated convolution. The FPN further incorporates PPM, Transer, and DenseFusion modules. The fine, medium, and coarse features have 64, 128, and 256 channels at 1/4, 1/16, and 1/32 resolution, corresponding to 160×160×64, 40×40×128, and 20×20×256 for a 640×640 image.

The multi-scale refinement query module integrates and correlates the extracted multi-scale feature maps to predict the matched acupoint ${\hat{p}}_{k}$ from the query point $q_{k}$, progressively improving localization accuracy across three granularities: coarse, medium, and fine. The multi-scale refinement query framework of COTR directly crops local image patches and repeatedly feeds the cropped images back into the network. In contrast, the proposed module uses feature-map cropping as a computationally efficient alternative to repeated image-domain cropping. The two operations are not assumed to be strictly equivalent because receptive fields, interpolation, contextual information, and boundary effects may differ. Nevertheless, feature-map cropping avoids repeated backbone inference while retaining local contextual information for coarse-to-fine refinement. Hence, the proposed module performs cropping directly on the feature maps centered at $q_{k}$ and ${\hat{p}}_{k}$, obtaining local feature regions for each target acupoint. Regardless of the acupoint or refinement stage, this strategy avoids repeated invocation of the feature extractor and accelerates multi-scale refinement.

**Figure 4:**
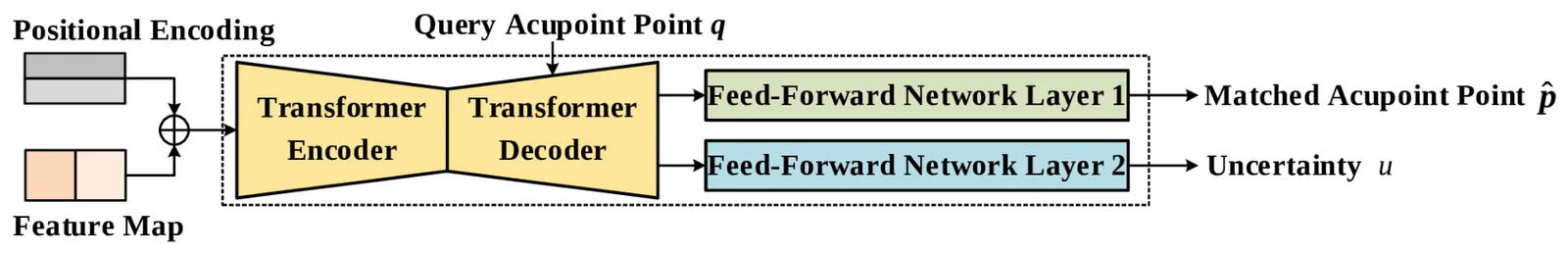
Transformer-basedmatching-inference pathway.

The module comprises three matching-inference pathways, one for each granularity, and each pathway consists of a Transformer and two feed-forward networks, as shown in Figure 4. The fine, medium, and coarse Transformers use embedding dimensions of 64, 128, and 256 and feed-forward dimensions of 128, 256, and 512, respectively. Each contains three encoder layers, three decoder layers, eight attention heads, a dropout rate of 0.1, and ReLU activation. The coarse stage uses the complete 1/32 feature map without local cropping, while the medium- and fine-stage feature-map crop sizes are 17×17 and 13×13, respectively. When a requested local crop extends beyond the valid feature-map boundary, its crop indices are clipped to the valid feature-map range before refinement. The Transformer encoder performs self-attention on each individual image and cross-attention between the TCM template image and the target hand image. Its input consists of feature maps augmented with linear positional encodings. For a pixel located at $s={\left[u,v\right]}^{T}$, the positional encoding PE(s) is expressed as

(1) $$P\left(s\right)=\left[p_{1}\left(s\right),p_{2}\left(s\right),\ldots ,p_{C/4}\left(s\right)\right],$$

(2) $$p_{i}\left(s\right)=\left[\sin\left(i\pi s^{T}\right),\cos\left(i\pi s^{T}\right)\right],$$

where *C* denotes the number of channels in the feature map. Each $p_{i}\left(s\right)$ contributes four components (sine and cosine terms for both *u* and *v*); therefore, PE(s) has dimension *C* and matches the feature-channel dimension.

The Transformer decoder receives the encoder output together with the position-encoded query point $q_{k}$. Because each hand acupoint is queried independently, self-attention among different query points is disabled. Two feed-forward networks then predict the matched acupoint location and its corresponding uncertainty; the latter is used to filter unreliable matches. To explicitly distinguish the correspondence direction, $F_{k,i}^{r}$ and $F_{k,i}^{t}$ denote the reference/template and target feature inputs, respectively, at refinement stage i∈{c,m,f}. The implemented forward matching takes the template query $q_{k}$ as input and predicts its corresponding target location ${\hat{p}}_{k,i}$. Thus, the forward template-to-target matching function is written as

(3) $$\left({\hat{p}}_{k,i},u_{k,i}\right)=M_{i}\left(F_{k,i}^{r},F_{k,i}^{t},q_{k}\right),\;i\in \left\{c,m,f\right\}.$$

Finally, the position and uncertainty of the *k*-th hand acupoint in the target hand image are expressed as

(4) $${\hat{p}}_{k}={\hat{p}}_{k,f},\;u_{k}=u_{k,c}+u_{k,m}+u_{k,f}.$$

The supervised correspondence loss is computed only in the forward template-to-target direction. The target ground-truth point $p_{k}$ is used only as supervision for the predicted target location ${\hat{p}}_{k,i}$, yielding

(5) $$L_{k,i}^{\mathrm{match}}={∥{\hat{p}}_{k,i}-p_{k}∥}_{2}^{2}.$$

For cycle consistency, the forward prediction ${\hat{p}}_{k,i}$ is subsequently used as a query for the reverse target-to-template operation. In this reverse operation, the order of the target and reference feature inputs is exchanged, and the recovered template point is defined as

$${\tilde{q}}_{k,i}=M_{i}\left(F_{k,i}^{t},F_{k,i}^{r},{\hat{p}}_{k,i}\right).$$

The cycle-consistency loss is then computed between the recovered template point and the original template query:

(6) $$L_{k,i}^{\mathrm{cycle}}={∥{\tilde{q}}_{k,i}-q_{k}∥}_{2}^{2}.$$

The reverse target-to-template operation is used solely to construct the cycle-consistency constraint and is not treated as an additional supervised matching direction. When uncertainty prediction is considered, the loss function for the *i*-th matching-inference module is expressed as

(7) $$L_{k,i}=\left(1-u_{k,i}\right)\left(L_{k,i}^{\mathrm{match}}+L_{k,i}^{\mathrm{cycle}}\right)+\lambda_{i}u_{k,i},$$

where $\lambda_{i}>0$ denotes the uncertainty regularization coefficient. The predicted uncertainty score $u_{k,i}$ is constrained to [0,1], with larger values indicating greater uncertainty. Hence, the optimization problem for the multi-scale refinement query module is formulated as

(8) $$\min\sum_{k=1}^{K}\left(L_{k,c}+L_{k,m}+L_{k,f}\right).$$

The problem is solved using the Adam optimizer. For uncertainty-based reliability filtering, the rejection threshold $\tau^{*}$ was selected exclusively on the validation set. A correspondence with a normalized localization error greater than 0.05 (the PCK@0.05 boundary) was treated as unreliable. Among candidate thresholds, $\tau^{*}$ was chosen to maximize the F1 score for identifying unreliable correspondences and was then fixed for test-set evaluation. The complete TCM-template-based acupoint localization procedure is summarized in Algorithm 1.
**Algorithm 1** TCM-template-based acupoint localization method**Input:** $I_{\mathrm{tmp}}$, $I_{q}$ and $\left\{q_{k}|k=1,2,\ldots ,K\right\}$**Output:** $\left\{{\hat{p}}_{k}|k=1,2,\ldots ,K\right\}$Obtain multi-scale feature maps of $I_{\mathrm{tmp}}$ and $I_{q}$ and fuse them into $F_{1/32}$, $F_{1/16}$, and $F_{1/4}$.**for** k=1 to *K* **do**   $F_{k,c}=F_{1/32}$, $\left({\hat{p}}_{k,c},u_{k,c}\right)=M_{c}\left(F_{k,c},q_{k}\right)$.   Crop $F_{k,m}$ from $F_{1/16}$ centered at ${\hat{p}}_{k,c}$, and compute $\left({\hat{p}}_{k,m},u_{k,m}\right)=M_{m}\left(F_{k,m},q_{k}\right)$.   Crop $F_{k,f}$ from $F_{1/4}$ centered at ${\hat{p}}_{k,m}$, and compute $\left({\hat{p}}_{k,f},u_{k,f}\right)=M_{f}\left(F_{k,f},q_{k}\right)$.   ${\hat{p}}_{k}={\hat{p}}_{k,f}$, $u_{k}=u_{k,c}+u_{k,m}+u_{k,f}$.   **if** $u_{k}>$ Threshold **then** delete ${\hat{p}}_{k}$; **end if****end for**

### 2.3. Datasets and Experimental Settings

#### 2.3.1. Hand-Acupoint Dataset Construction and Annotation

The hand-acupoint dataset used in this study was constructed from two sources: a screened subset of the publicly available 11K Hands dataset [16] and laboratory-collected volunteer hand images. Specifically, 870 images were selected from 11K Hands, and the remaining 530 images were acquired from 160 volunteer participants using smartphone RGB cameras. Camera models and native image resolutions varied across devices, and illumination, background, acquisition distance, and hand pose were not fixed. The primary acquisition requirement was that the hand region and visible hand surface were sufficiently clear for subsequent physician annotation. After image screening and quality control, 693 palm images and 707 dorsal-hand images were retained for subsequent annotation, model training, validation, and internal evaluation. The composition of the hand-acupoint dataset is summarized in Table 1.

Institutional ethics approval was not required for the laboratory image acquisition because the study involved only non-invasive acquisition and analysis of anonymized hand images and did not involve clinical intervention, diagnosis, treatment, or patient medical data, in accordance with the applicable institutional requirements. Written informed consent was obtained from all volunteer participants before image acquisition. The participants were informed that their anonymized hand images would be used for hand-acupoint dataset construction, physician annotation, algorithm training and evaluation, academic publication, and editorial or peer-review verification where necessary. No directly identifiable personal information was used in this study.

**Figure 5:**
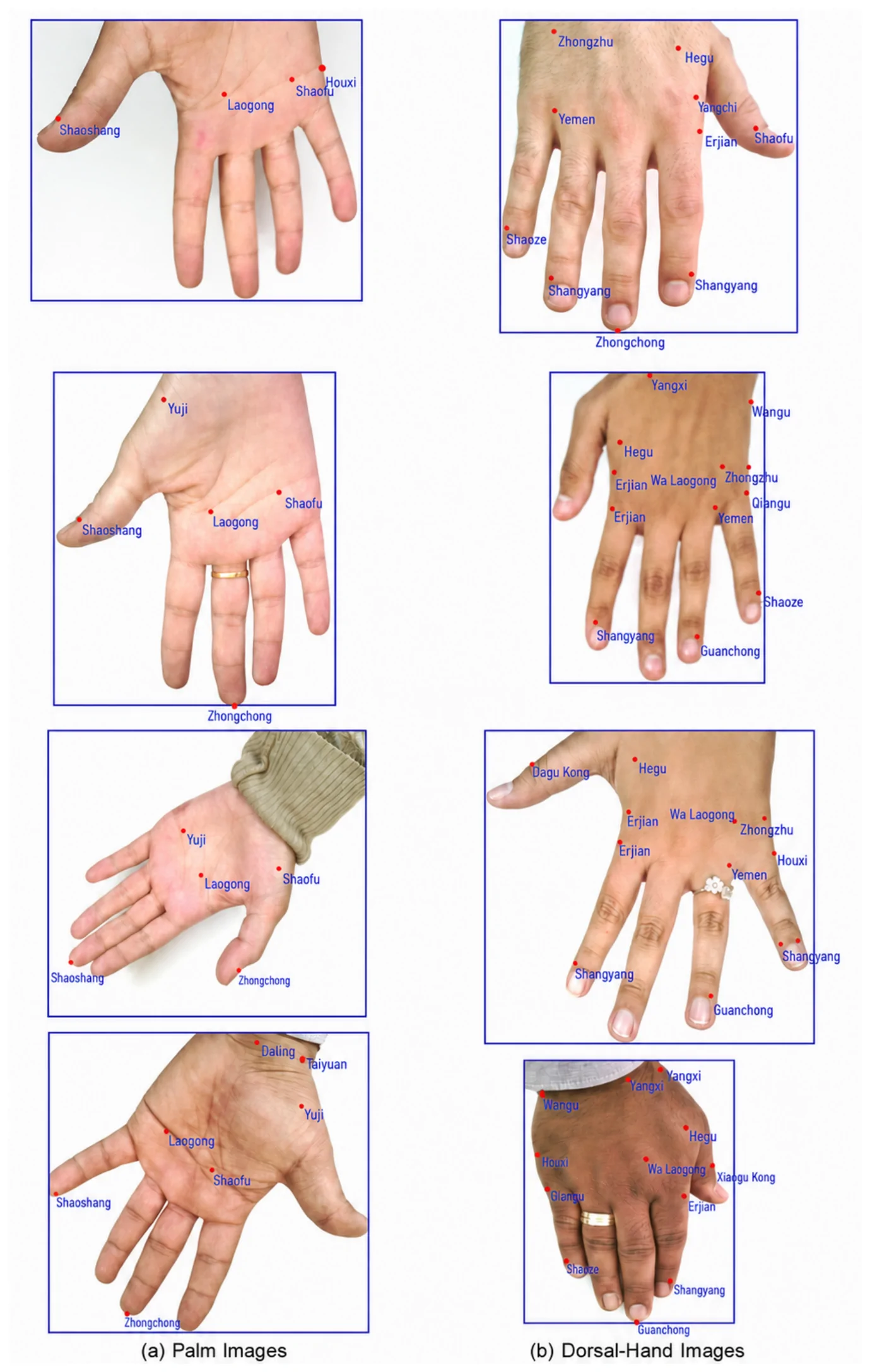
Sample visualization of the acupoint keypoint dataset. Chinese labels denote the corresponding traditional Chinese medicine acupoint names; red points indicate physician-annotated acupoint locations, and blue rectangles delineate the annotated hand.

Senior physicians from the Seventh Affiliated Hospital of Sun Yat-sen University manually annotated the hand-acupoint key points. Only clearly visible and determinable acupoints in each view were annotated. Acupoints that were outside the image field of view, severely occluded, or difficult to determine from the visible hand surface were treated as missing labels and were excluded from the corresponding loss and metric calculation. As shown in Table 2 and Table 3, 7 palm-side acupoints and 21 dorsal-hand acupoints were annotated. Representative physician-annotated palm and dorsal-hand samples from the constructed acupoint keypoint dataset are shown in Figure 5. Some edge acupoints, such as Zhongchong, may appear in both palm and dorsal-hand images depending on the viewpoint. The sample count in Table 2 and Table 3 therefore refers to the number of annotated image instances for each acupoint rather than the number of individuals. Hand bounding boxes were also provided for all images and were used only to define the scale normalization in quantitative evaluation. The annotated ground-truth bounding boxes were not used to crop test images and were not supplied as model inputs during test inference.

The 11K Hands images were incorporated into the constructed hand-acupoint dataset after screening and physician annotations. They were not used as an independent external-validation dataset; accordingly, all quantitative results reported in this study represent internal evaluation rather than cross-dataset validation.

Dataset split and registration pair generation.The dataset contains 1400 images from 378 participants. To avoid data leakage caused by image-pair generation, the dataset was first divided at the participant level into training, validation, and test subsets at an approximately 7:2:1 ratio. The resulting subsets contained 265, 75, and 38 participants, corresponding to 980, 280, and 140 images, respectively. Images from the same participant were assigned exclusively to one subset. Sparse image-registration pairs were subsequently generated independently within each partition; thus, neither participants nor images were shared across the training, validation, and test subsets.

**Figure 6:**
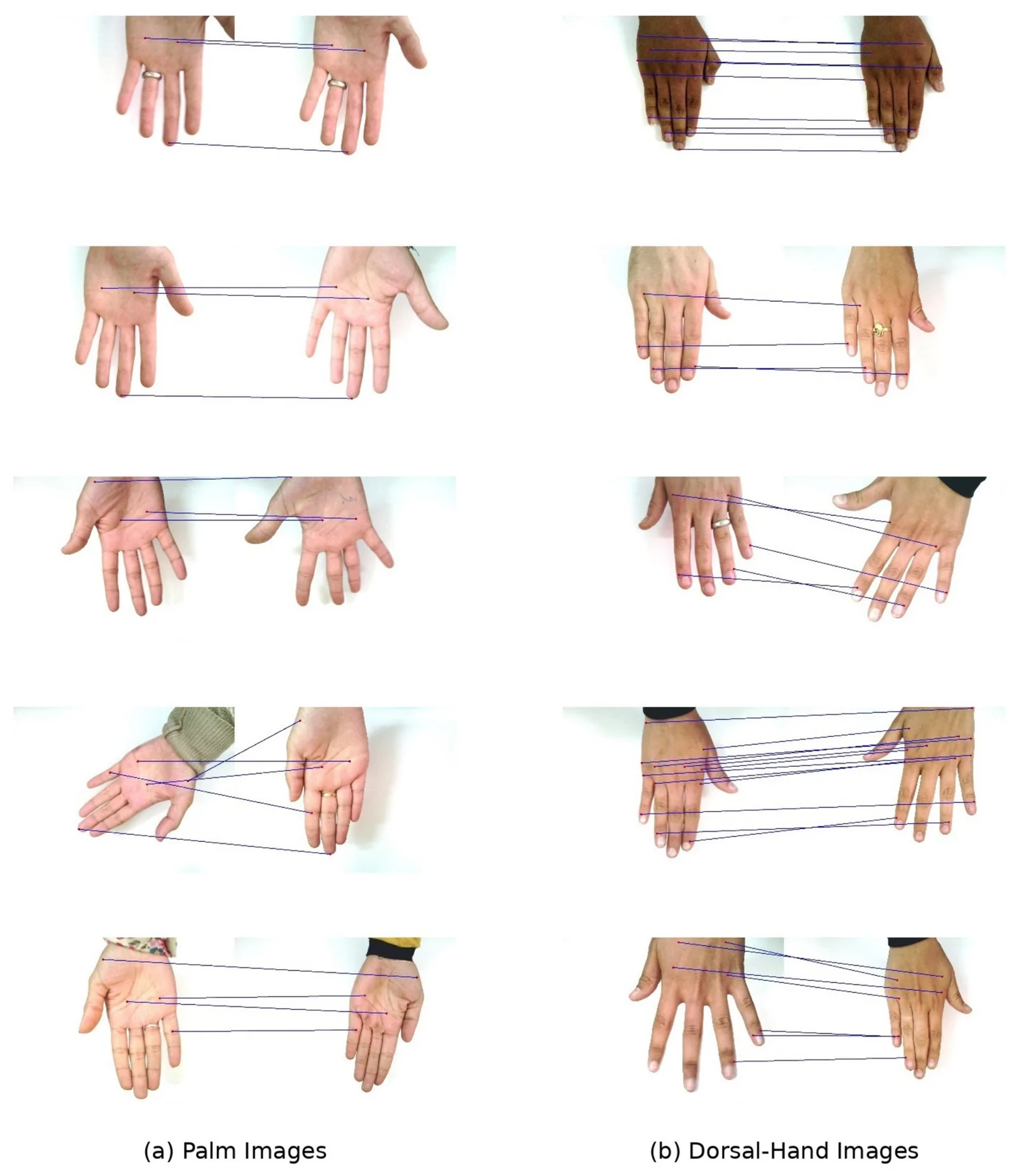
Sample visualization of the acupoint sparse image-registration dataset.

Within each subset, self-pairs and cross-view pairs were excluded. Image pairs were generated only between images from the same hand view, namely palm-to-palm or dorsal-to-dorsal pairs. For each image pair, only acupoints visible and annotated in both images were retained as valid sparse correspondences, and pairs without shared annotated acupoints were excluded. One image in each registration pair was treated as the template image and the other as the target image. The annotated acupoint coordinate in the template image was used as the query point, whereas the corresponding coordinate in the target image was used as the ground-truth matched point. This procedure yielded 2512, 737, and 368 registration pairs for the training, validation, and test subsets, respectively, containing 8280, 2424, and 1201 valid acupoint correspondences. Detailed statistics are provided in Table 4, and sample registration pairs are shown in Figure 6.

#### 2.3.2. Experimental Protocol and Evaluation Metrics

To validate the effectiveness and superiority of the proposed marker-free hand acupoint localization framework, we compare it with keypoint-detection-based methods, including YOLOv8-pose [10,11] and YOLOv11-pose [17], and with the COTR model [14]. For the keypoint approaches, two separate models are trained on the palm and dorsal-hand keypoint datasets, respectively, because the fixed number of predicted key points cannot accommodate the different sets and counts of acupoints on each side. In contrast, the proposed sparse-registration method is trained on the unified sparse image-registration dataset, requiring only a single model. Three metrics are adopted for comparison.

The first metric is Average Acupoint Positioning Error (AAPE), which measures the mean Euclidean localization error between the predicted acupoint coordinates and the physician-annotated ground-truth coordinates. Let *N* denote the total number of valid evaluated acupoint instances rather than the number of acupoint categories; $p_{i}$ and ${\hat{p}}_{i}$ are the ground-truth and predicted positions of the *i*-th valid acupoint instance, respectively. AAPE is defined as

(9) $$\mathrm{AAPE}=\frac{1}{N}\sum_{i=1}^{N}{∥{\hat{p}}_{i}-p_{i}∥}_{2}.$$

The second metric is Percentage of Correct Keypoints (PCK@threshold), which measures localization accuracy under a normalized error threshold. Let $\sigma_{i}$ denote the normalization factor for the *i*-th acupoint instance, defined as the diagonal length of the corresponding hand bounding box. A prediction is considered correct under PCK@α when its Euclidean localization error is less than or equal to α times the hand bounding-box diagonal. PCK is given by

(10) $$\mathrm{PCK}@\mathrm{threshold}=\frac{1}{N}\sum_{i=1}^{N}\delta \left(\frac{{∥{\hat{p}}_{i}-p_{i}∥}_{2}}{\sigma_{i}}\right),$$

(11) $$\delta \left(x\right)=\left\{\begin{array}{cc} 1, & x\leq \mathrm{threshold},\\ 0, & \mathrm{otherwise}. \end{array}\right.$$

In this study, the normalized thresholds were set to 0.01, 0.03, and 0.05 to evaluate localization under increasingly relaxed normalized-error tolerances.

The third metric is Average Inference Time (AIT), which quantifies the mean processing time under the same hardware environment and evaluation protocol for all compared methods.

For keypoint-detection methods, the loss weights for hand-detection bounding-box regression, classification, and acupoint localization are set to 7.5, 0.5, and 12.0, respectively. The initial learning rate, momentum, and weight-decay coefficients are 0.01, 0.937, and 0.0005. Both YOLOv8-pose and YOLOv11-pose were trained for 500 epochs with a batch size of 64. For the proposed sparse-registration method, Adam optimization was used with a fixed learning rate of $10^{-4}$ for 30 epochs and a batch size of 1; the Adam coefficients were $\beta_{1}=0.9$, $\beta_{2}=0.999$, and $\epsilon =10^{-8}$. No fixed random seed was specified in the original training configuration; the participant-level split itself was fixed and reused across the experiments. Training used fixed epochs without early stopping, and the checkpoint with the lowest validation loss was selected for test-set evaluation. The uncertainty regularization coefficients $\lambda_{i}$ for the coarse, medium, and fine scales were set to 0.1, 0.05, and 0.01, respectively; these values were empirically selected according to validation-set performance and fixed before test-set evaluation. The uncertainty rejection threshold $\tau^{*}$ was selected exclusively on the validation set and fixed for test-set evaluation. The ResNet50-FPN backbone was constructed without ImageNet initialization, and the model was subsequently initialized using pretrained correspondence-model checkpoint weights. Training images were resized to 640×640 pixels, whereas evaluation used 832×832 inputs after 32-pixel alignment. OpenCV bilinear interpolation was used for image resizing, and FPN upsampling used bilinear interpolation with align_corners=True. For a concatenated template-target image of width 2W and height *H*, a template coordinate (x,y) was normalized as (x/(2W),y/H) and a target coordinate as (0.5+x/(2W),y/H), so that template and target horizontal coordinates lie in [0,0.5] and [0.5,1], respectively, while the vertical coordinate lies in [0,1]. The query $q_{k}$, target ground truth $p_{k}$, and predictions ${\hat{p}}_{k,i}$ use this same convention. For local refinement, the horizontal coordinate is first mapped back to the corresponding single-image domain ($x_{\mathrm{local}}=2x$ for the template and $x_{\mathrm{local}}=2x-1$ for the target), and a coordinate within a local patch is normalized as (x−c)/s+0.5, where c and s denote the patch center and size. Final predictions are inverse-normalized and mapped back to the original-image coordinates. For inference, the batch size was 1. AIT was measured as the mean end-to-end inference time per target image for obtaining the complete set of acupoint predictions, including preprocessing and data transfer. Training and evaluation were conducted on a single NVIDIA A100-PCIE-40GB GPU. For the component-ablation analysis, all configurations use the same participant-level data split and evaluation protocol as the full model; only the component under study is changed. For the computational-complexity analysis, model parameters, FLOPs, and peak GPU memory were profiled under the corresponding evaluation configurations. FLOPs therefore reflect the actual evaluated implementations rather than a resolution-normalized architecture-only comparison. Wall-clock training time was not included in the cross-model comparison because it was not consistently recorded for all baseline methods under an identical profiling protocol; retrospective estimates were avoided. The augmentation protocols were separated by model type. For the YOLO-based keypoint-detection baselines, brightness adjustment, horizontal flipping, cropping, scaling, and mosaic augmentation were used. For the sparse-registration model, brightness adjustment and paired geometric augmentations (horizontal flipping, cropping, and scaling) were applied consistently to the template and target images, while mosaic augmentation was not used because it would disrupt the pairwise correspondence geometry. Acupoint coordinates and hand bounding boxes were transformed by the same geometric operation as the image; acupoints falling outside a valid cropped region were marked as missing and excluded from the corresponding loss.

For statistical comparison, paired bootstrap resampling is performed on the test set. Target images are resampled with replacement for 10,000 iterations while preserving the paired predictions of the compared methods. The 95% confidence intervals (CIs) of the paired differences in AAPE and PCK@0.05 are calculated separately for palm and dorsal-hand images. An improvement is considered statistically supported when the corresponding 95% CI does not include zero.

## 3. Results

### 3.1. Marker-Free Hand Acupoint Localization Experiments

Three evaluation metrics were used to compare the performance of all models, as shown in Table 5 and Table 6. For the proposed method, the reported localization results were calculated after applying the uncertainty rejection threshold $\tau^{*}$ selected on the validation set. For PCK, the normalized error thresholds were set to 0.01, 0.03, and 0.05 to evaluate localization at increasingly relaxed normalized-error tolerances. Dorsal-hand acupoints consistently achieved better localization than palm acupoints, which may be related to clearer visual structures and lower ambiguity in the dorsal-hand images. On the same hand datasets, keypoint-detection methods leverage the lightweight YOLO backbone and, therefore, achieve faster inference. YOLOv11-pose outperforms YOLOv8-pose by yielding lower average acupoint error and higher PCK across all thresholds. In contrast, sparse-registration approaches exploit the prior knowledge embedded in TCM-template images and rely on a Transformer-based cross-attention mechanism between template and target hand images. Consequently, they achieve higher localization accuracy than keypoint-detection methods on the internally constructed dataset, but at the cost of inference speed. By eliminating redundant feature-extraction operations, the proposed framework reduces inference time from 11,000 ms to 600 ms, corresponding to an approximately 94.5% reduction compared with COTR, while still fusing multi-scale features and slightly outperforming COTR in localization accuracy.

**Figure 7:**
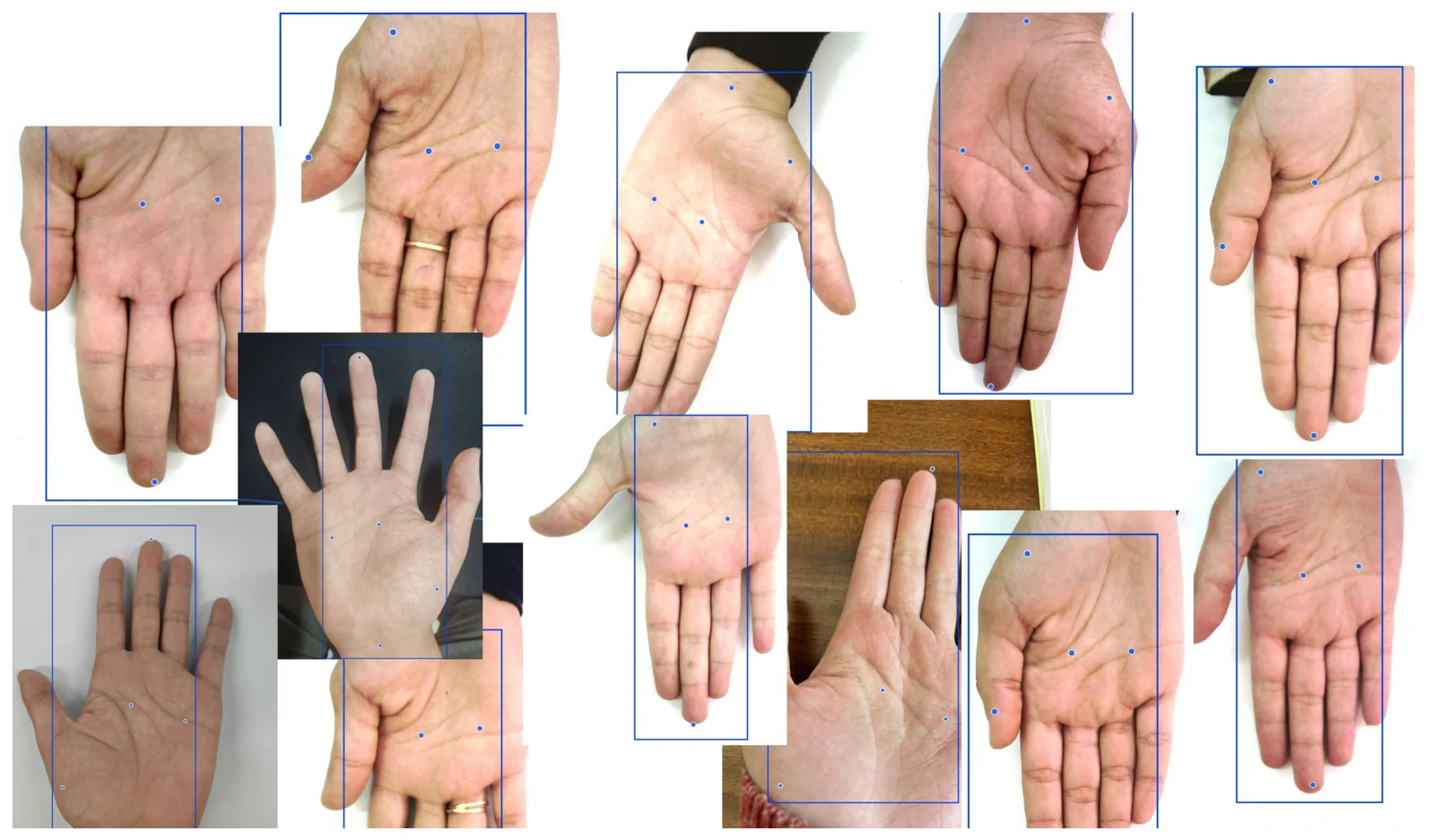
Representative palm acupoint localization results of the proposed method. Blue dots indicate the predicted acupoint locations, and blue rectangular boxes indicate the hand bounding boxes.

**Figure 8:**
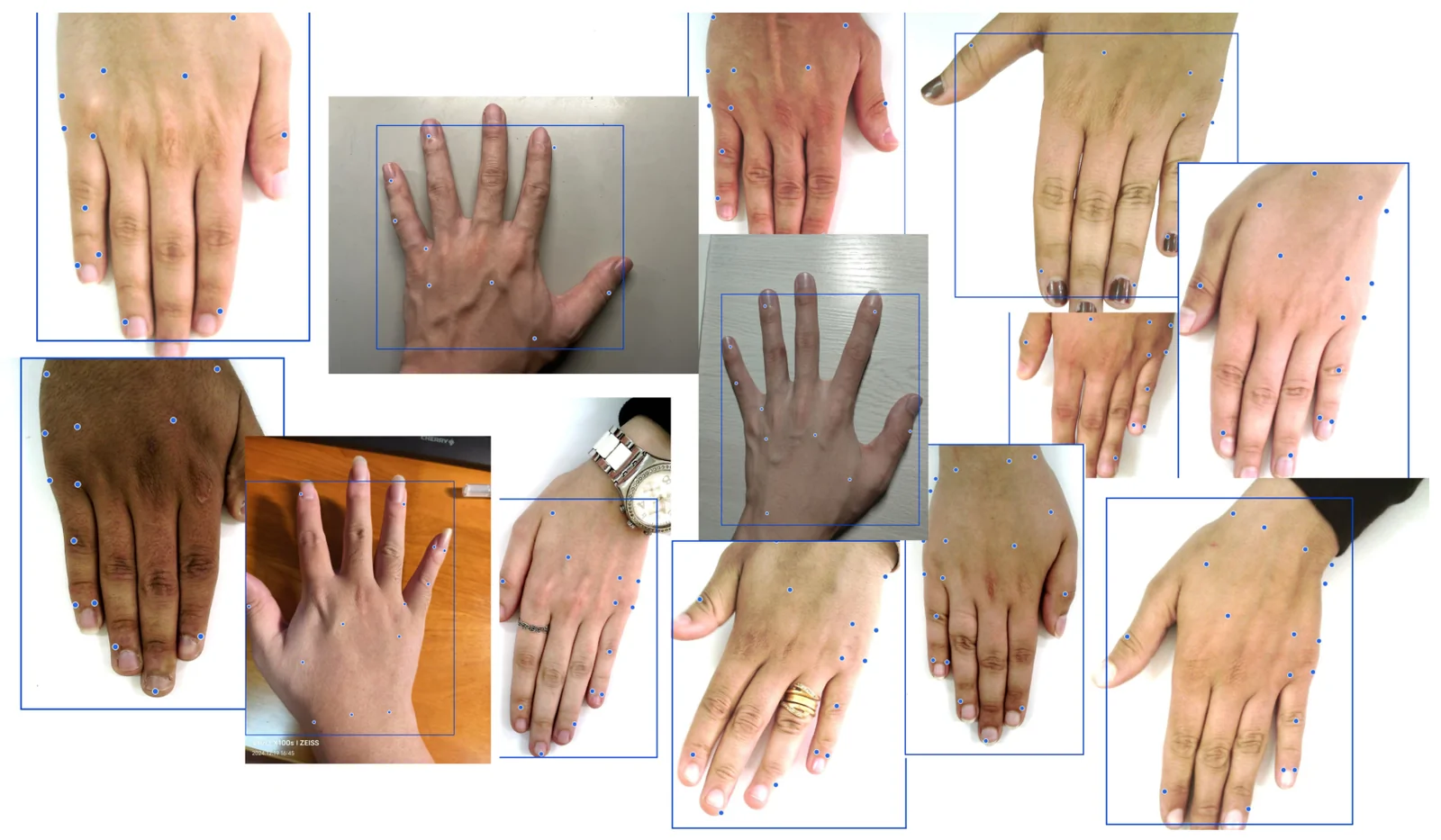
Representative dorsal-hand acupoint localization results of the proposed method. Blue dots indicate the predicted acupoint locations, and blue rectangular boxes indicate the hand bounding boxes.

As shown in Table 7, the YOLO-based keypoint detectors are substantially more compact than the registration-based methods. The proposed model has 31.44 M parameters, 283.08 GFLOPs, and a peak GPU-memory usage of 9.31 GB, reflecting the additional cost of multi-scale feature processing and Transformer-based correspondence refinement. Compared with COTR, the proposed architecture has higher static model complexity and memory use. Its inference-time advantage reported in Table 5 and Table 6 should therefore be attributed to reducing repeated feature-extraction operations during coarse-to-fine correspondence refinement rather than to a smaller network architecture. Static FLOPs alone do not capture this repeated-inference overhead. Representative localization results of the proposed method are shown in Figure 7 and Figure 8 for palm and dorsal-hand images, respectively. The blue dots indicate the predicted acupoint locations, while the blue rectangular boxes indicate the hand bounding boxes. These examples illustrate the localization outputs of the proposed sparse-registration method across hand images with variations in hand pose, scale, illumination, and background.

**Figure 9:**
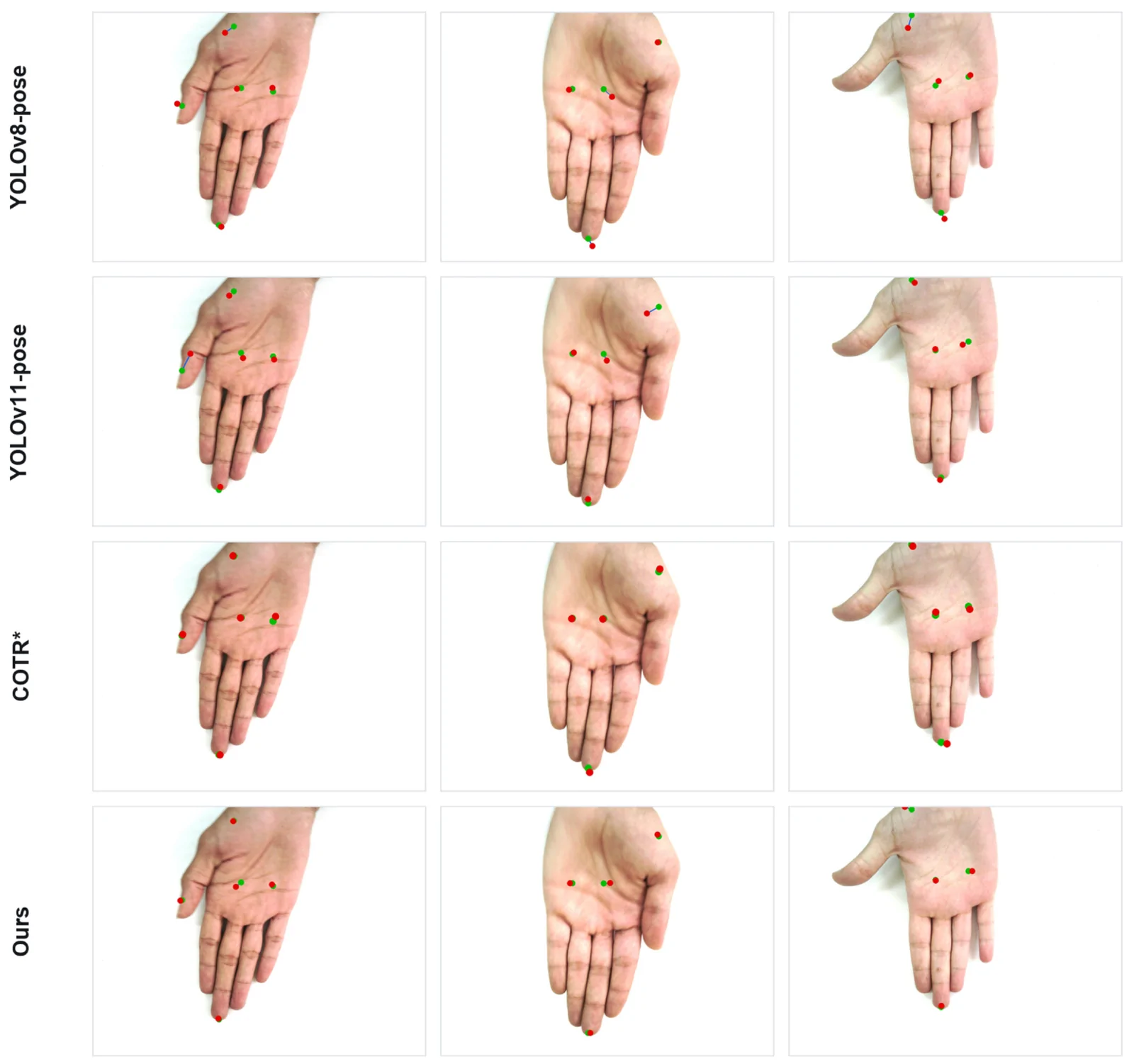
Visualization of palm acupoint localization comparisons. Green dots indicate the physician-annotated ground-truth acupoint locations, whereas red dots indicate the predicted acupoint locations produced by the evaluated models.

**Figure 10:**
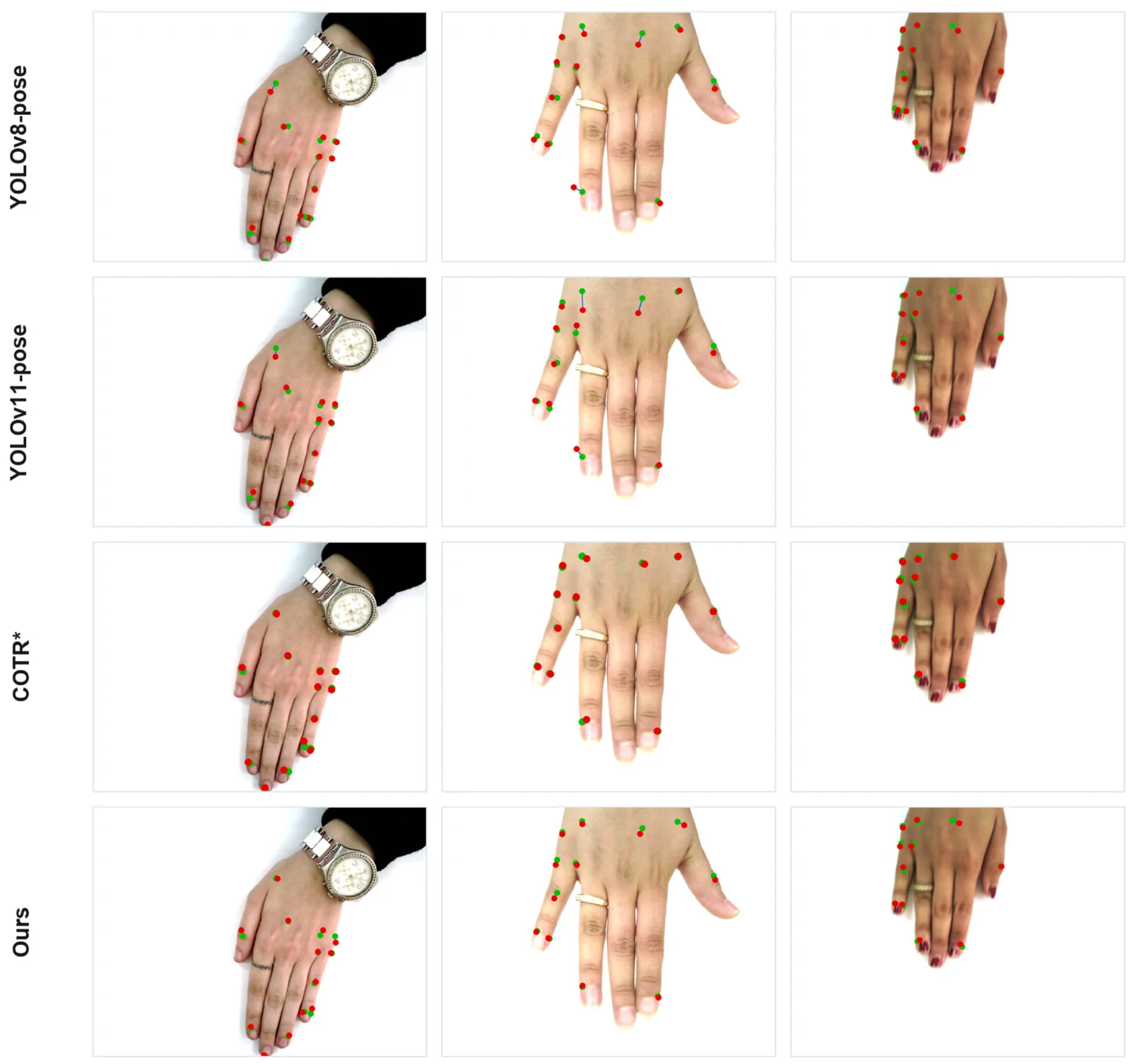
Visualization of dorsal-hand acupoint localization comparisons. Green dots indicate the physician-annotated ground-truth acupoint locations, whereas red dots indicate the predicted acupoint locations produced by the evaluated models.

Sample visualizations of hand-acupoint localization are presented in Figure 9 and Figure 10, where green and red dots denote the ground-truth and predicted locations, respectively, and matching pairs are linked by blue lines. For acupoints near the wrist, keypoint-detection methods yield low-confidence estimates that often fall outside the image field of view, causing detection failure. Likewise, edge acupoints frequently extend beyond the hand region, leading to localization errors.

### 3.2. Ablation and Reliability Analyses

#### 3.2.1. Component Ablation

To isolate the contribution of the principal design choices, component ablations are performed under the same data split, training protocol, and evaluation settings as the full model. The multi-scale refinement is evaluated progressively using the coarse stage alone, the coarse–medium stages, and the complete coarse–medium–fine pipeline. The efficiency contribution of feature-map cropping is evaluated against image-domain cropping with repeated feature extraction while keeping the remaining matching architecture unchanged. Cycle consistency and uncertainty weighting are evaluated by removing each component individually from the full model. The ablation configurations and evaluation metrics are summarized in Table 8.

The progressive refinement stages reduce AAPE from 30.84 to 18.42 pixels for palm images and from 21.63 to 12.60 pixels for dorsal-hand images. Image-domain cropping yields localization accuracy close to the feature-map implementation but increases AIT from 600 ms to 2180 ms, supporting the efficiency benefit of avoiding repeated feature extraction. Removing cycle consistency or uncertainty weighting degrades localization accuracy in both views.

#### 3.2.2. Uncertainty Reliability Analysis

The reliability of the learned uncertainty is evaluated independently of the threshold-selection procedure. Spearman’s rank correlation between the calibrated uncertainty score and normalized localization error is positive for both palm (ρ=0.61) and dorsal-hand (ρ=0.57) predictions, indicating that larger uncertainty scores tend to be associated with larger localization errors. For the calibrated uncertainty scores used in threshold selection, the validation-set distribution had $u_{\min}^{\mathrm{cal}}=0.00438$, $u_{\mathrm{median}}^{\mathrm{cal}}=0.01051$, and $u_{\max}^{\mathrm{cal}}=0.74988$. Predictions with normalized localization error greater than 0.05 were treated as unreliable during validation. Maximizing the validation-set F1 score yielded a maximum F1 of 0.790 and a rejection threshold of $\tau^{*}=0.010938$. This threshold was then fixed for test-set evaluation, with predictions whose calibrated uncertainty exceeded $\tau^{*}$ rejected as unreliable. The before/after rejection performance and retained prediction coverage are summarized in Table 9.

#### 3.2.3. Statistical Significance Analysis

To quantify the statistical support for the reported improvements, paired bootstrap comparisons are performed between the proposed method and each baseline using the procedure described above. The analysis is conducted separately for palm and dorsal-hand images, and the 95% CIs of the paired differences in AAPE and PCK@0.05 are summarized in Table 10.

## 4. Discussion

The experimental results show that template-guided sparse image registration achieves higher localization accuracy than the selected keypoint-detection baselines on the evaluated hand-acupoint dataset. In direct keypoint detection, the network must learn the appearance and spatial distribution of every acupoint directly from annotated samples. In contrast, the proposed template-query strategy embeds expert prior knowledge into the localization process: the annotated TCM template provides explicit query points, and the network focuses on estimating their correspondences in the target hand image. By forming valid same-view registration pairs from the images, this formulation provides additional correspondence supervision from the same image set without claiming sample-efficiency beyond the evaluated dataset.

The proposed method also provides a practical route for deep-learning-based sensor medical image analysis. The input is a camera-acquired hand image rather than a manually attached marker or a complex multimodal sensing signal, which makes the framework convenient for non-contact image acquisition. Rather than claiming clinical standardization, the present results indicate that template-guided sparse registration may provide a technical basis for more consistent image-based acupoint annotation under the evaluated conditions. Compared with COTR, the feature-map-level refinement strategy avoids repeated image cropping and redundant backbone inference. This explains the large reduction in inference time while maintaining or improving localization accuracy and makes the framework relevant to sensor-based medical imaging applications in which both accuracy and computational efficiency are important.

Beyond standalone localization, the proposed method may also serve as an image-based perception component within TCM teaching and rehabilitation-training systems. Recent studies have explored cross-view multimodal vision for assessment of TCM rehabilitation training [18], mobile natural human–robot interaction for virtual acupuncture teaching [19], and mixed-reality acupuncture training environments [20]. In these settings, reliable acupoint localization could provide spatial references for visual guidance, training feedback, and alignment with virtual or robotic interfaces. Accordingly, the present method is better regarded as a potential localization module that can complement such assessment and training frameworks, rather than as a standalone clinically validated system. Future work will investigate system-level integration and user-oriented validation in these educational and rehabilitation scenarios.

Dorsal-hand acupoints achieved better localization performance than palm acupoints. One possible reason is that dorsal-hand images contain more stable anatomical structures, such as finger joints and metacarpal contours, whereas palm images are more affected by skin creases, texture variation, and pose-dependent deformation. Edge and wrist acupoints remain more difficult because they may appear near the image boundary or outside the dominant hand region, which can reduce the reliability of both keypoint detection and template-based matching.

Several limitations should be noted. First, the relatively limited dataset size introduces a potential risk of overfitting. To reduce this risk, participant-level separation was performed before image-pair generation, and data augmentation was applied during training. The annotation distribution is also imbalanced across acupoints because some fingertip, wrist, and edge acupoints are not visible or determinable in all views. Second, the volunteer images were acquired under unconstrained conditions using different smartphone cameras, with variations in image resolution, illumination, background, acquisition distance, and hand pose. This diversity provides some variation in the evaluated data; however, the present study did not perform stratified evaluations by illumination condition, camera device, skin color, or hand pose. Therefore, generalization across these factors remains to be established using larger and more diverse external datasets. Third, the current study evaluates technical agreement with physician-provided image annotations; it does not establish clinically validated acupoint localization or evaluate physical placement accuracy on the body surface, treatment outcomes, usability, or procedural safety. Fourth, the method may depend on the selected template, the pose and perspective of the target image, and the consistency of expert annotations. Fifth, the Transformer-based matching module is still slower than lightweight YOLO-pose models, although it is substantially faster than COTR. The current inference speed is more appropriate for offline or semi-interactive annotation assistance than for fully real-time deployment with multiple templates. An independently collected and physician-annotated external dataset was not available for the present study; therefore, cross-dataset generalization has not yet been quantitatively validated. Future work should include participant-level external validation, more diverse acquisition conditions, repeated annotation by multiple experienced TCM practitioners, uncertainty calibration, device-bias analysis, and model compression for more efficient deployment.

## 5. Conclusions

This paper proposes a marker-free hand-acupoint localization method based on deep feature correspondence learning and sparse image registration for sensor-based medical image analysis. The designed sparse-registration network learns acupoint-level correspondences between a TCM-template image and a camera-acquired target hand image, enabling target acupoints to be estimated under the guidance of TCM prior knowledge. A Transformer architecture strengthens cross-image feature association, and a multi-scale refinement query module progressively improves localization accuracy while avoiding redundant feature extraction. Experiments on the internally constructed physician-annotated palm and dorsal-hand datasets support improved image-based localization performance over the selected baselines under the evaluated conditions. In future work, we will increase dataset diversity, include additional body regions after further validation, perform external and participant-independent validation, and explore more robust and efficient deep-learning models for sensor-based acupoint localization.

## Figures and Tables

**Table 1:** Composition of the hand-acupoint image dataset.

Source	Number of Images	Role in This Study
Screened subset of 11K Hands	870	Retained from 218 participants and used after physician annotation. Up to four views per participant were retained, covering left/right palm and dorsal views.
Laboratory-collected volunteer images	530	Acquired from 160 volunteer participants, with 2–4 images per participant, under unconstrained smartphone-based conditions; used after written informed consent and physician annotation.
Total	1400	378 participants; used for model training, validation, and internal evaluation.

**Table 2:** Annotation status of palm acupoint key points.

Acupoint	Location	Sample Count
*Shaofu* (HT8)	Between the 4th and 5th metacarpal bones, at the site touched by the tip of the little finger when a fist is made.	679
*Laogong* (PC8)	Between the 2nd and 3rd metacarpal bones (closer to the 3rd), at the site touched by the tip of the middle finger when a fist is made.	683
*Yuji* (LU10)	At the midpoint of the radial side of the 1st metacarpal bone, at the junction of the red and white skin (thenar eminence).	601
*Zhongchong* (PC9)	At the midpoint of the tip of the middle finger.	637
*Shaoshang* (LU11)	On the radial side of the distal phalanx of the thumb, 0.1 cun proximal to the corner of the nail.	133
*Daling* (PC7)	At the midpoint of the transverse crease of the wrist, between the tendons of the palmaris longus and the flexor carpi radialis.	231
*Taiyuan* (LU9)	On the radial side of the volar wrist crease, in the depression over the radial artery pulsation.	232

**Table 3:** Annotation status of dorsal-hand acupoint key points.

Acupoint	Location	Sample Count
*Shaoze* (SI1)	On the radial side of the distal phalanx of the little finger, 0.1 cun proximal to the nail corner.	558
*Shangyang* (LI1)	On the radial side of the distal phalanx of the index finger, 0.1 cun proximal to the nail corner.	700
*Guanchong* (TE1)	On the radial side of the distal phalanx of the ring finger, 0.1 cun proximal to the nail corner.	696
*Sanjian* (LI3)	On the radial side of the 2nd metacarpal bone, in the depression just posterior to the metacarpal head.	169
*Zhongzhu* (TE3)	In the depression between the 4th and 5th metacarpal bones.	695
*Erjian* (LI2)	On the radial side of the 2nd metacarpal bone, at the distal junction of the red-and-white skin.	153
*Qiangu* (SI2)	On the red-and-white margin at the level of the metacarpophalangeal crease anterior to the 5th metacarpophalangeal joint.	475
*Houxi* (SI3)	On the radial side of the 5th metacarpal bone, at the distal transverse palmar crease where the red-and-white skin meets.	467
*Shaochong* (HT9)	On the radial side of the distal phalanx of the little finger, 0.1 cun proximal to the nail corner.	690
*Hegu* (LI4)	Midway between the 1st and 2nd metacarpal bones, on the radial side of the midpoint of the 2nd metacarpal bone.	629
*Wailaogong* (Ex-UE8)	Between the 2nd and 3rd metacarpal bones, 0.5 cun posterior to the metacarpophalangeal joint.	701
*Yemen* (TE2)	Between the 4th and 5th metacarpal bones, posterior to the web margin at the red-and-white skin junction.	705
*Dagukong* (Extra)	At the midpoint of the 1st intermetacarpal space.	674
*Wangu* (SI4)	In the depression between the base of the 5th metacarpal bone and the hamate bone.	201
*Zhongchong* (PC9)	At the midpoint of the tip of the middle finger.	244
*Yangxi* (LI5)	On the dorsal distal transverse wrist crease, in the depression anterior to the radial styloid process.	243
*Xiaogukong* (EX-UE6)	At the midpoint of the 5th intermetacarpal space.	684
*Yanggu* (SI5)	On the dorsal wrist crease at the radial aspect, in the depression anterior to the ulnar head.	151
*Zhongkui* (EX-UE4)	At the midpoint of the first phalanx of the middle finger, proximal to the palm.	698
*Yangchi* (TE4)	On the dorsal distal transverse wrist crease, in the depression on the ulnar side of the common extensor tendon.	281
*Zhongquan* (EX-UE3)	Between the 2nd and 3rd metacarpal bones, 0.5 cun posterior to the metacarpophalangeal joint.	261

**Table 4:** Statistics of the dataset partitions and generated sparse-registration pairs.

View	Images (Train/Val/Test)	Registration Pairs (Train/Val/Test)	Valid Correspondences (Train/Val/Test)
Palm	485/139/69	948/281/135	2056/599/298
Dorsal	495/141/71	1564/456/233	6224/1825/903
Total	980/280/140	2512/737/368	8280/2424/1201

**Table 5:** Comparative table of palm acupoint localization performance.

Method	AAPE (Pixels)	PCK@0.01	PCK@0.03	PCK@0.05	AIT (ms)
YOLOv8-pose	45.92	0.0842	0.6460	0.9307	45.92
YOLOv11-pose	32.90	0.1413	0.7065	0.9348	47.94
COTR	24.40	0.2261	0.8728	0.9969	11,000
Ours	18.42	0.3448	0.9766	1.0000	600

**Table 6:** Comparative table of dorsal-hand acupoint localization performance.

Method	AAPE (Pixels)	PCK@0.01	PCK@0.03	PCK@0.05	AIT (ms)
YOLOv8-pose	27.57	0.1903	0.8158	0.9548	45.39
YOLOv11-pose	24.30	0.2469	0.8628	0.9699	46.56
COTR	15.09	0.4203	0.9920	1.0000	11,000
Ours	12.60	0.5528	0.9982	1.0000	600

**Table 7:** Computational-complexity comparison of the evaluated methods. Parameters, FLOPs, and peak GPU memory were measured under the corresponding evaluation configurations.

Method	Params (M)	FLOPs (G)	Peak GPU Memory (GB)
YOLOv8-pose	3.30	9.18	0.21
YOLOv11-pose	2.87	7.47	0.26
COTR	18.39	207.09	5.08
Ours	31.44	283.08	9.31

**Table 8:** Component ablation results for the proposed sparse-registration method.

View	Variant	Refinement	Cropping Strategy	Cycle	Uncertainty	AAPE	PCK@0.01	PCK@0.03	PCK@0.05	AIT
Palm	Coarse only	C	N/A	Yes	Yes	30.84	0.2015	0.8472	0.9724	310
Palm	Coarse + medium	C + M	Feature-map	Yes	Yes	23.17	0.2816	0.9318	0.9917	445
Palm	Image-domain cropping	C + M + F	Image-domain + re-extraction	Yes	Yes	18.16	0.3512	0.9774	1.0000	2180
Palm	w/o cycle consistency	C + M + F	Feature-map	No	Yes	21.36	0.3067	0.9528	0.9958	585
Palm	w/o uncertainty weighting	C + M + F	Feature-map	Yes	No	19.74	0.3275	0.9683	0.9989	575
Palm	Full model	C + M + F	Feature-map	Yes	Yes	18.42	0.3448	0.9766	1.0000	600
Dorsal	Coarse only	C	N/A	Yes	Yes	21.63	0.3541	0.9298	0.9867	310
Dorsal	Coarse + medium	C + M	Feature-map	Yes	Yes	15.82	0.4687	0.9841	0.9981	445
Dorsal	Image-domain cropping	C + M + F	Image-domain + re-extraction	Yes	Yes	12.43	0.5590	0.9983	1.0000	2180
Dorsal	w/o cycle consistency	C + M + F	Feature-map	No	Yes	14.51	0.4894	0.9926	0.9991	585
Dorsal	w/o uncertainty weighting	C + M + F	Feature-map	Yes	No	13.37	0.5278	0.9963	1.0000	575
Dorsal	Full model	C + M + F	Feature-map	Yes	Yes	12.60	0.5528	0.9982	1.0000	600

**Table 9:** Uncertainty rejection analysis on the test set. The rejection threshold $\tau^{*}=0.010938$ was selected exclusively on the validation set by maximizing the F1 score and was fixed for test-set evaluation.

View	Setting	Coverage	AAPE (Pixels)	PCK@0.01	PCK@0.03	PCK@0.05
Palm	Before rejection	1.0000	22.73	0.2924	0.9415	0.9768
Palm	After rejection	0.8240	18.42	0.3448	0.9766	1.0000
Dorsal	Before rejection	1.0000	15.48	0.4876	0.9794	0.9912
Dorsal	After rejection	0.8460	12.60	0.5528	0.9982	1.0000

**Table 10:** Paired bootstrap comparison between the proposed method and the baseline methods. Negative ΔAAPE and positive ΔPCK@0.05 favor the proposed method.

View	Comparison	ΔAAPE (95% CI)	ΔPCK@0.05 (95% CI)
Palm	Ours vs. YOLOv8-pose	−27.50 [−34.82, −20.61]	+0.0693 [0.0361, 0.1017]
Palm	Ours vs. YOLOv11-pose	−14.48 [−20.06, −9.12]	+0.0652 [0.0327, 0.0958]
Palm	Ours vs. COTR	−5.98 [−9.36, −2.71]	+0.0031 [0.0000, 0.0092]
Dorsal	Ours vs. YOLOv8-pose	−14.97 [−19.31, −10.76]	+0.0452 [0.0196, 0.0708]
Dorsal	Ours vs. YOLOv11-pose	−11.70 [−15.88, −7.75]	+0.0301 [0.0091, 0.0507]
Dorsal	Ours vs. COTR	−2.49 [−4.73, −0.61]	0.0000 [0.0000, 0.0000]

## Data Availability

The physician-annotated hand-image and acupoint-annotation data generated or curated in this study are available from the corresponding author upon reasonable request and subject to privacy and consent restrictions related to human hand images. The publicly available 11K Hands dataset should be accessed through its original source.
